# Main causes of death of free-ranging bats in Turin province (North-Western Italy): gross and histological findings and emergent virus surveillance

**DOI:** 10.1186/s12917-023-03776-0

**Published:** 2023-10-11

**Authors:** Elena Colombino, Davide Lelli, Sabrina Canziani, Giuseppe Quaranta, Cristina Guidetti, Stefania Leopardi, Serena Robetto, Paola De Benedictis, Riccardo Orusa, Mitzy Mauthe von Degerfeld, Maria Teresa Capucchio

**Affiliations:** 1 Department of Veterinary Sciences, Centro Animali Non Convenzionali (C.A.N.C), University of Turin, Turin, Italy; 2 Istituto Zooprofilattico Sperimentale della Lombardia e Dell’Emilia Romagna, Brescia, Italy; 3 Molecular Medicine PhD Program, Department of Medicine and Surgery, University of Parma, Parma, Italy; 4 Liguria e Valle d’Aosta, Istituto Zooprofilattico Sperimentale del Piemonte, National Reference Centre for Wild Animal Diseases (CeRMAS), Aosta, Italy; 5 Istituto Zooprofilattico Sperimentale delle Venezie, FAO and National Reference Centre for Rabies, Legnaro, PD Italy

**Keywords:** Bat, Necropsy, Emergent viruses, Pathology, Histology

## Abstract

**Background:**

Bats are recognized as reservoir species for multiple viruses. However, little is known on bats’ health and mortality. Thus, this study aimed to investigate the main causes of death of bats from Turin province (North-western Italy) and to describe gross and histopathological lesions potentially associated with the presence of selected bat viruses.

**Results:**

A total of 71 bats belonging to 9 different species of the families *Vespertilionidae* and *Molossidae* were necropsied and samples of the main organs were submitted to histopathological examination. Also, aliquots of the small intestine, liver, spleen, lung, and brain were collected and submitted to biomolecular investigation for the identification of *Coronaviridae, Poxviridae, Reoviridae* (Mammalian orthoreovirus species), *Rhabdoviridae* (*Vaprio ledantevirus* and *Lyssavirus* species) and *Kobuvirus*. The majority of bats died from traumatic lesions due to unknown trauma or predation (n = 40/71, 56.3%), followed by emaciation (n = 13/71,18.3%). The main observed gross lesions were patagium and skin lesions (n = 23/71, 32.4%), forelimbs fractures (n = 15/71, 21.1%) and gastric distension (n = 10/71,14.1%). Histologically, the main lesions consisted of lymphoplasmacytic pneumonia (n = 24/71, 33.8%), skin/patagium dermatitis (n = 23/71, 32.4%), liver steatosis and hepatitis (n = 12, 16.9%), and white pulp depletion in the spleen (n = 7/71, 9.8%). Regarding emergent bat viruses, only *poxvirus* (n = 2, 2.8%) and *orthoreovirus* (n = 12/71, 16.9%) were detected in a low percentage of bats.

**Conclusions:**

Trauma is the main lesion observed in bats collected in Turin province (North-western Italy) associated with forelimb fractures and the detected viral positivity rate seems to suggest that they did not represent a threat for human health.

**Supplementary Information:**

The online version contains supplementary material available at 10.1186/s12917-023-03776-0.

## Introduction

Bats (order Chiroptera) make up more than 20% of extant mammals and inhabit every continent except Antarctica. They accounted for more than 1400 species divided into two suborders, *Yinpterochiroptera* and *Yangochiroptera* [1]. Among European species, at least 44 species have been recorded in Europe and 34 in Italy [2].

The role of Chiroptera as reservoirs for multiple infectious agents had been confirmed by the increasing identification of viruses in a wide range of bat species around the world, also thanks to the next-generation sequencing technology [3]. Particularly, viruses detected in bats belong to different families, including *Adenoviridae, Astroviridae, Coronaviridae, Picornaviridae, Poxviridae, Reoviridae* and *Rhabdoviridae* [4]. Moreover, bats were confirmed as important reservoir hosts for zoonotic viruses, among which lyssaviruses (several species), henipaviruses (Hendra virus and Nipah virus) and Marburg virus induce severe diseases in humans. In addition, bats carry other viruses, such as coronaviruses and ebolaviruses, that are phylogenetically correlated with highly pathogenic human viruses [3, 5–7]. Such a wide genetic diversity of bat viruses is likely related to the parallel diversity of bat species but it might be also due to the unique ecological characteristics of bats, including high population sizes, crowded roosting behaviours and seasonal habits syncronized across populations [8].

Among the above-mentioned viruses, members of the *Coronaviridae, Poxviridae, Reoviridae*, and *Rhabdoviridae* families, along with Kobuviruses can potentially represent a threat for the human health and they were identified in the Italian territory [9–12]. Firstly, Alpha and betacoronaviruses have been frequently reported in bats [13, 14] and in Italy the phylogenetic analysis of RNA-dependent RNA polymerase sequence fragments identified several coronaviruses from *Pipistrellus kuhlii*, *Rhinolophus hipposideros*, *Nyctalus noctula*, and *Hypsugo savii* [14]. Particularly, *an Hypsugopoxvirus was* isolated in Italy in 2019 from an *Hypsugo savi* bat and even if these viruses are phylogenetically dissimilar and have diverse clinical impacts on their hosts, its viral ecology and disease associations should be better investigated [15]. Secondly, O*rthoreoviruses have been* frequently reported in bats in Europe and Italy mainly in *Pipistrellus khulii species*, suggesting that further investigations are needed to evaluate their relationship with human strains [10, 16, 17].

Thirdly, Rhabdoviruses are frequently found in bats worldwide. In Europe, rabies-related lyssaviruses are currently the only proven zoonotic pathogens founds in bats, even though reports of other Lyssavirus species are also available [18].

Finally, *Kobuvirus* genus including *Aichivirus*, are well-known enteric pathogens in humans and they have been recently sequenced in a *Pipistrellus pipistrellus* in Italy, suggesting their presence also in European bats [9].

Although many studies have been published on different viruses detected in bats, little attention has been given to bats’ health probably due to their unique immune system and physiology which can prevent the osbervation of overt diseases after viral infections. To date, in Europe, only Mühldorfer et al. [19, 20] and Hajkova et al. [21] studied the disease susceptibility, infection rates, and the associated lesions in free-ranging bats in Germany and Czech Republic. Thus, only a few studies described histological lesions associated to viral infection in European bats (Table 1).

This can be due to the small size of European bats that undergo a very quickly carcass decomposition, representing a limit for anatomopathological examination. Moreover, gross lesions are rarely evident and histopathological examination becomes essential for a comprehensive evaluation [20].

Thus, this study aimed to investigate the main causes of death of bats from Turin province (North-western Italy) and to describe gross and histopathological lesions potentially associated with the presence of selected bat viruses [9–11, 13, 15].

**Table 1:** Histopathological lesions associated with viral infection in bats

Family	Genus	Organs/Samples from which virus has been isolated	Reported histopathological lesions	References
*Filoviridae*	*Ebolavirus*	Brain, liver, oral/rectal swabs, lung and spleen	Non suppurative pneumonia; depletion of lymphoid follicles in spleen	[22]
*Herpesviridae*	*Rhadinovirus*	Lung, liver, spleen, kidney	Pneumonia	[23]
*Reoviridae*	*Orthoreovirus*	Lung, intestine, liver, spleen	Hemorrhagic or catarrhal-hemorrhagic enteritis, pneumonia, activation of the lymphoreticular tissue in the spleen	[10, 16, 17, 24]
*Togaviridae*	*Alfavirus*	Blood, organ pool	Non-significant lesions	[25]

## Results

### Animals

The 71 necropsied bats belonged to 9 species, family *Vespertilionidae* and *Molossidae* (Additional file 1). The overall sex ratio was 45 males (63.4%) and 26 females (36.6%). Regarding age, the majority of the analysed bats were adults (n = 29/71, 40.8%), followed by subadults (n = 28/71, 39.4%), juveniles (n = 8/71 11.3%) and neonates (n = 6/71, 8.5%). Overall, bats with less than 1 year (neonates, juveniles, subadults) represented more than a half (59.1%) of all the examined bats. Moreover, the majority of the bats were rescued in summer (n = 48/71, 67.6%) and in autumn (n = 13/71, 18.3%).

**Fig. 1:**
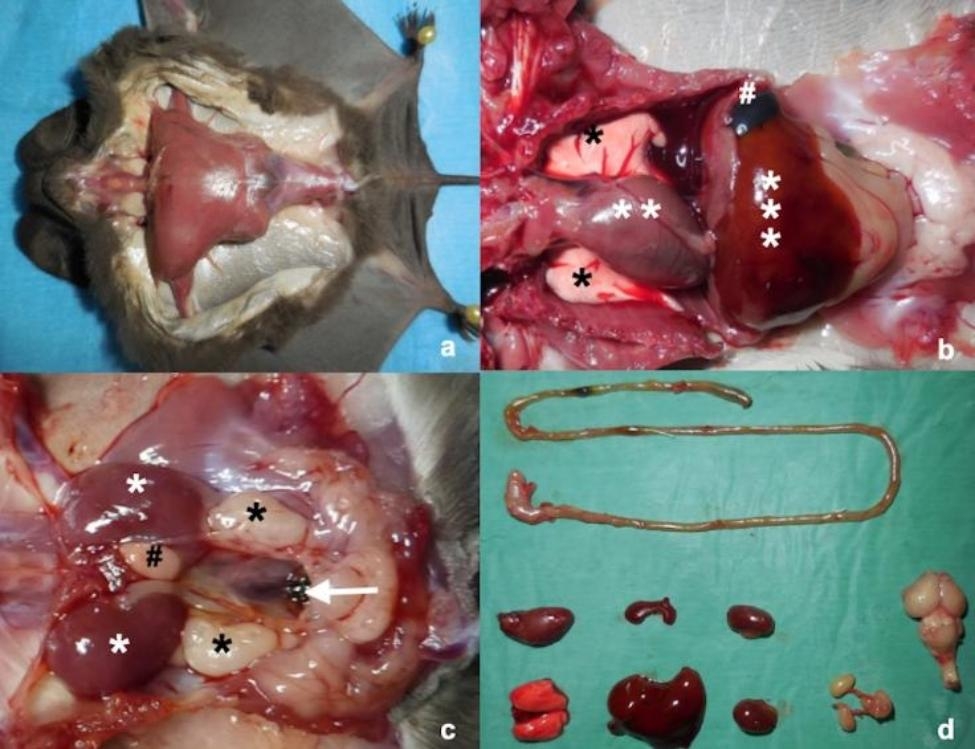
Main steps of bat necropsy (*Tadarida teniotis*, adult, male). **a**) Pinning the bat to a sterile board to facilitate the necropsy and skinning along the ventral midline to visualize the pectoral muscles and the abdominal wall. **b**) Abdominal and thoracic cavities opened by incising the abdominal wall along the ventral midline and the costal margins and by incising the ribs on both sides of the sternum. Lungs (black asterisk) and heart (double white asterisks) are easily detectable in the thoracic cavity. The diaphragm divides the abdominal viscera (liver-triple white asterisks-spleen-#- and gastro-intestinal tract covered by omentum) from the thorax. **c**) Particular of the urogenital system. From top to bottom: kidneys (white asterisk), adrenal gland (#), testicle (black asterisks), urinary bladder and genital glands laterally to the urinary bladder (white arrow). **d**) All the viscera removed and individually examined. From the top, first line: gastro- intestinal tract. Second line from left to the right: heart, spleen, kidney and brain. Third line from left to the right: lung, liver, kidney, testicle with deferens and urinary bladder

### Necropsy and histopathological findings

**Fig. 2:**
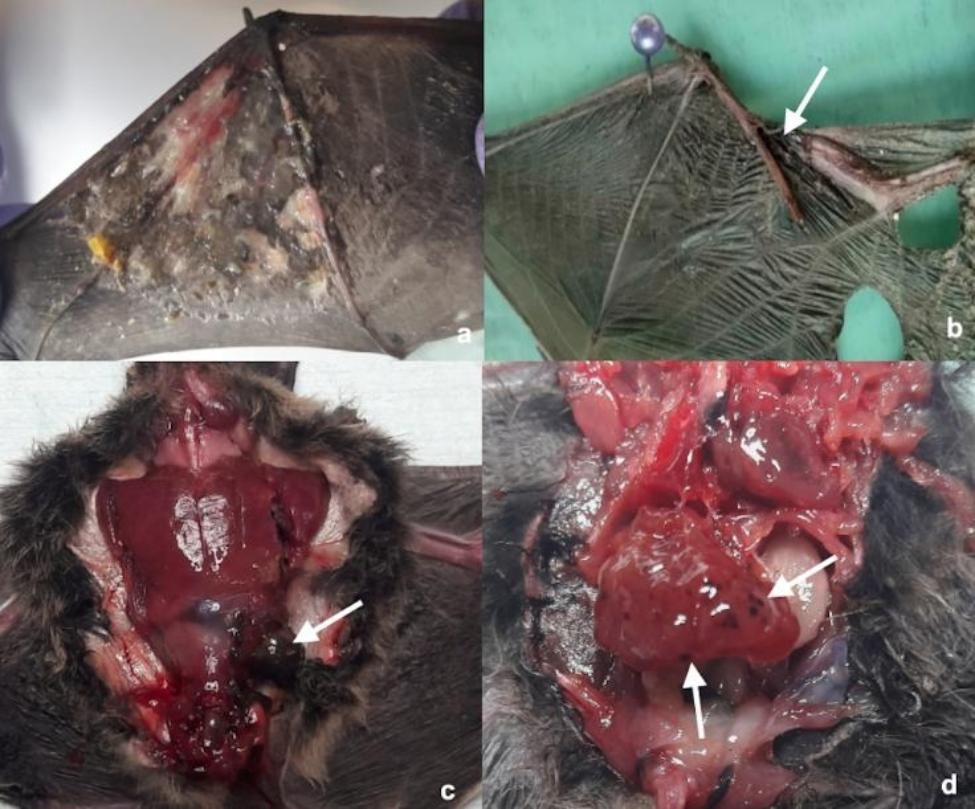
Main macroscopical traumatic findings recorded in the bat of the present study. **a**) *Hypsugo savii*, male, subadult. Severe and diffuse dermatitis of the patagium. **b**) *Pipistrellus khulii*, male, adult. Multiple patagium laceration and radio-ulnar fracture (white arrow). **c**) *Pipistrellus khulii*, male, subadult. Laceration of the left lateral abdominal wall with partial gastric herniation (white arrow). **d**) *Pipistrellus khulii*, male, subadult. Multiple liver petechiae

**Fig. 3:**
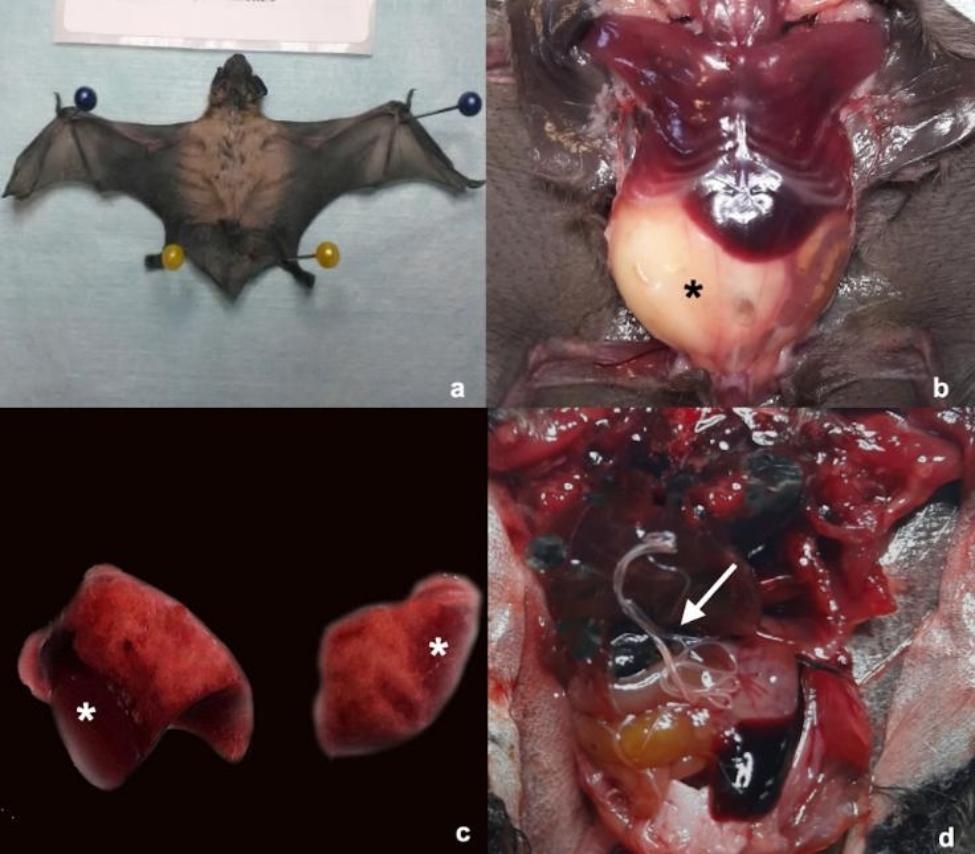
Main macroscopical abdominal and toracic findings recorded in the bat of the present study. **a**) *Hypsugo savii*, male, neonate. Severe emaciation. **b**) *Pipistrelluskhulii*, male, subadult. Severe gastric distension (black asterisk). **c**) *Hypsugo savii*, female, juvenile. Moderate and multifocal pneumonia (white asterisk). **d**) *Hypsugo savii*, male, juvenile. *Litosomoides* spp. infestation in the abdominal cavity

Additional file 2 summarized the main causes of death and associated gross post-mortem findings of the examined bats. About 56.3% of bats showed traumatic lesions due to unknown trauma (n = 35/71) or predation (n = 5/71), mainly represented by patagium and skin lesions (n = 23/71, 32.4%) (Fig. 2a), fractures (n = 15/71, 21.1%) (Fig. 2b), diaphragmatic hernias (n = 1/71; 1.4%) (Fig. 2c) and liver petechiae with abdominal haemorrhages (n = 1/71, 1.4%) (Fig. 2d). Particularly, radio-ulnar fractures (n = 7/71, 9,0.8%), open and close fractures of the humerus (n = 3/71, 4.2%), phalanges fractures or ablation (n = 3/71, 4.2%) and fractures of the carpus (n = 2/71, 2.8%) were recorded. Thirteen bats died for severe emaciation (18.3%) and they were all rescued in summer (Fig. 3a) while for 18 bats out of 71 the cause of death cannot be established (25.4%). In addition to these lesions, gastric distension (n = 10/71, 14.1%) (Fig. 3b) was mainly recorded in summer (n = 5/10, 50.0%) and autumn (n = 4/10, 40.0%). Moreover, pneumonia (n = 3/71, 4.2%) (Fig. 3c), free nematodes in thorax and/or abdomen (*Litomosoides* spp.) (n = 4/71, 5.6%) (Fig. 3d) and spleen decolouration (n = 1/71, 1.4%) were also observed.

**Fig. 4:**
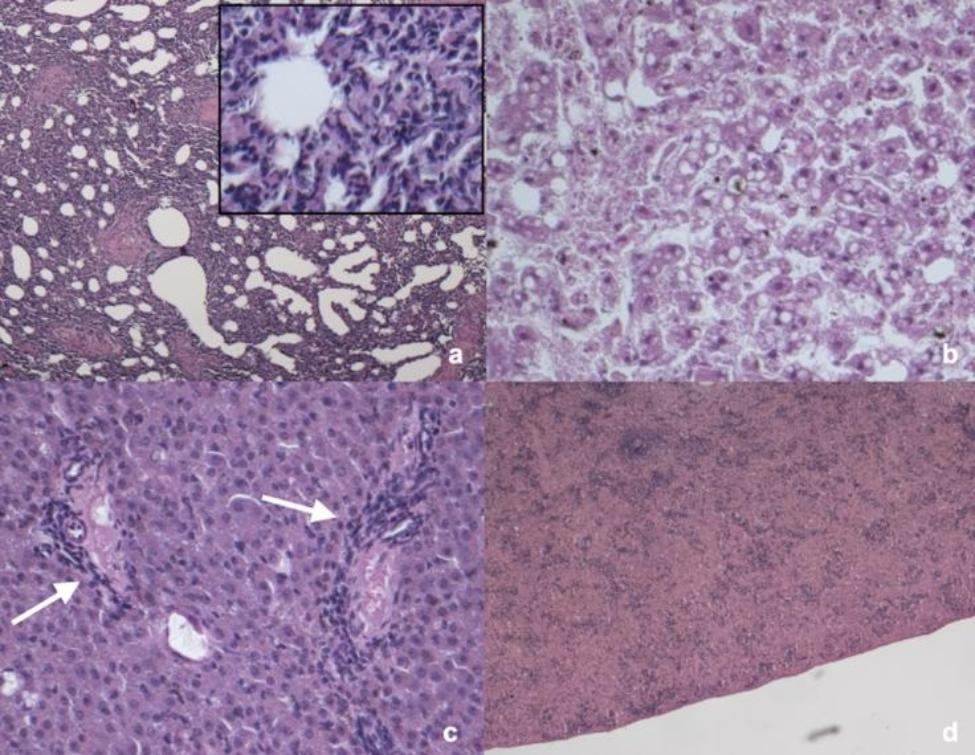
Main histological findings recorded in lung, liver and spleen of the bats of the present study. **a**) *Pipistrellus khulii*, male, juvenile. Lung, moderate and diffuse lymphoplasmacytic interstitial pneumonia, Haematoxilyn and eosin (H-e), 5x. Inset: higher magnification of the lymphoplasmacytic infiltrates, H-e, 10x. **b**) *Tadarida teniotis*, male, adult. Liver, moderate and multifocal steatosis, H-e, 20x. **c**) *Pipistrellus khulii*, male, adult. Liver,mild and multifocal lymphoplasmacytic hepatitis (white arrows), H-e, 20x. **d**) *Hypsugo savii*, male, subadult. Spleen. Moderate and diffuse white pulp depletion, H-e, 5x

**Fig. 5:**
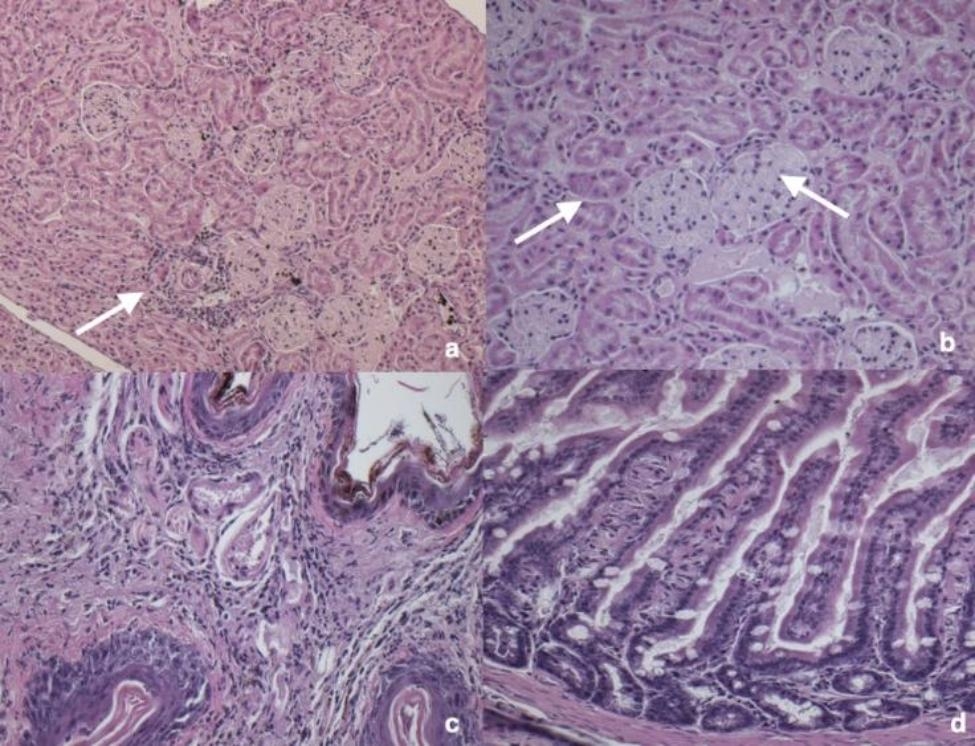
Main histological findings recorded in kidney, patagium and small intestine of the bats of the present study (*Hypsugo savii*, male, adult). **a**) Kidney, mild and multifocal interstitial nephritis (white arrows), H-e, 10x. **b**) Kidney, mild and multifocal membranous glomerulopathy (white arrows), H-e, 20x. **c**) Patagium, severe and diffuse lymphoplasmacytic dermatitis, H-e, 20x. **d**) Small intestine, mild and multifocal lymphoplasmacytic enteritis, H-e, 20x

Histopathological alterations were summarized in Additional file 3. Lung was predominantly affected (n = 24/71, 33.8%), mainly showing mild to moderate, multifocal to diffuse interstitial pneumonia (n = 19) characterized by lymphoplasmacytic infiltrates (Fig. 4a) and mild to moderate lymphoplasmacytic bronchiolitis with bronchus-associated lymphoid tissue (BALT) activation (n = 5). Twelve bats presented histological lesions in the liver: mild to severe multifocal steatosis was recorded in 4 bats (Fig. 4b), mild to moderate multifocal lymphoplasmacytic hepatitis was present in 6 bats (Fig. 4c) while 2 bats showed both mild to severe multifocal steatosis and lymphoplasmacytic hepatitis. Particularly, liver steatosis was mainly recorded in bats rescued in summer (n = 3/10, 30.0%), followed by spring (n = 1/10, 10.0%) and autumn (n = 1/10, 10.0%). Of the 10 bats with splenic lesions, 7 presented moderate and diffuse white pulp depletion (Fig. 4d), one showed moderate and diffuse red pulp depletion while two presented severe and diffuse necrosis with macrophage activation. In kidneys, mild and multifocal tubular steatosis (n = 1), mild and multifocal interstitial nephritis (n = 1) (Fig. 5a), and mild and multifocal membranous glomerulopathy were recorded (n = 1) (Fig. 5b). All the patagium and skin with macroscopical lesions were characterized by acute to chronic dermatitis with different grades of hyperkeratosis and infiltrates of lymphocytes, macrophages and scattered neutrophils (Fig. 5c). No positivity for periodic acid Schiff/Grocott’s- Gomori staining was detected in the patagium/skin samples. In three intestines, mild and multifocal lymphoplasmacytic enteritis was recorded (Fig. 5d). Non-significant alterations were recorded in the brain and heart of all the examined bats.

### Biomolecular and virological investigations

Coronaviruses, Kobuviruses, Lyssaviruses, and *Vaprio ledantevirus* were not detected in the examined bats. On the contrary, MRV were detected in 12 bats out of 71 (16.9%) collected in summer (n = 10/71, 14.1%) and autumn (n = 2/71, 2.8%). Particularly, 6 bats showed PCR positivity for MRV in the intestine, 3 bats resulted MRV positive both in the intestine and pool of viscera and the remaining three bats resulted MRV positive in all the collected samples (intestine, lungs, pool of viscera). Moreover, 6 were Savi’s pipistrelles (*Hypsugo savii*) and 5 were Kuhl’s pipistrelles (*Pipistrellus khulii*); 4 bats had less than 1 year and 8 were adults. Males (n = 6/12, 50.0%) and females (n = 6/12, 50.0%) were equally affected. All the MRV positive samples were isolated on cell culture and confirmed by genome sequencing (OR339562, OR339563, OR339564, OR339565, OR339566, OR339567, OR339568, OR339569, OR339570, OR339571, OR339572). The remaining samples resulted negative by in-vitro cell culture isolation. Eleven out of twelve MRV positive samples have been typed as MRV serotype 3 through the specific S1 RT-PCR. These MRV-positive animals showed heterogenous microscopical lesions in liver (n = 4/71, 5.6%), lung (n = 2/71, 2.8%), spleen (n = 1/71, 1.4%) and kidney (n = 1/71, 1.4%).

Also, 2 bat’s lungs out 71 tested positive for *Poxvirus* isolation (2.8%) in cell culture and then confirmed by PCR and genome sequencing (OR339573 and OR339574). Both bats belonged to the *H.savii* species, they were less than one year of age, one male and one female and they were rescued in summer. Interestingly, the two bats positive for *Poxvirus* showed a mild and multifocal lymphoplasmacytic pneumonia. The results of the BLAST analysis showed the highest nucleotides sequence identities to *Hypsugopoxvirus*, a poxvirus previously detected in *Hypsugo savii* bats (strain 251170-23/2017- GenBank accession number MK860688.1). The similarity ranged from 98.69 to 99.09 with a query cover of 100%.

## Discussion

To the author’s knowledge, this is the first study evaluating the main causes of death, gross and histopathological findings in free-ranging bats of Turin province (North-western Italy). The findings of this study highlighted that the majority of free-ranging bats seemed to die from trauma or emaciation, being male adult (n = 29/71, 40.8%) or subadult (n = 28, 39.4%) bats the most affected categories. Histologically, the main lesions consisted of pneumonia, skin/patagium dermatitis, liver steatosis and hepatitis, and white pulp depletion in the spleen. Regarding emergent bat viruses, only Poxvirus and Orthoreovirus were detected in the bats of the present study (2.8% and 16.9%, respectively).

Particularly, 56.3% of the free-ranging bats died or were euthanized after a severe trauma. This is in accordance with previous studies conducted in Germany and Czech Republic, which reported that almost three-quarters of all bat deaths in Europe can probably be attributed to trauma or cat predation [20, 26, 27]. Moreover, patagium lacerations (n = 23/71, 32.4%) and forelimb fractures (n = 15/71, 21.1%) were the most frequent traumatic lesions. Similarly, Mühldorfer et al. [20] reported that about 90% of the observed fractures in bats were in the upper extremities. The second most reported cause of death in the present study was emaciation in absence of any sign of trauma (n = 13/71, 18.3%). Emaciation can be a consequence of chronic illness or extreme hunger and in bats, it can be due to low body temperature if they are disturbed during hibernation in winter [28]. However, in the present study, emaciation was recorded in bats rescued in summer and it can be probably linked to chronic illness or the high temperature recorded in Turin province during this season.

Interestingly, gastric distension is the third most common macroscopical finding of this study (n = 10/71, 14.1%). Particularly, in neonates and juveniles’ bats (less < 1 year) this can be attributed to the incorrect feeding management or overfeeding of bats. In fact, at necropsy their stomach was full of milk and it has been already reported that there is a tendency to overfeed bats in captivity [29]. This procedure, particularly in neonates, promote gastric distention with the following shock due to cardiac failure. Moreover, the adult bats also shown multiple traumatic lesions and the gastric distension could be consequent to a neurological damage.

Regardless of the causes of death and the macroscopic lesions, house-roosting bats including Savi’s pipistrelles (*Hypsugo savii*) and Kuhl’s pipistrelles (*Pipistrellus kuhlii*) approached 87.3% of the total deceased bats and they were mainly brought to the rescue centre in summer (n = 48/71, 67.6%). These results reflected the presence of synanthropic species found near human settlements and it is in accordance with the previous work by Ancillotto et al. [30]. In fact, *Hypsugo savii* and *Pipistrellus kuhlii are* the most common bats in the urban areas of Italy; they are sedentary and they roost in buildings, foraging in a wide range of habitats [2]. Also, the over-representation of house roosting bats in the sample could be due to the fact that CANC received only bats rescued by people, an event definitely more likely to occur near houses and in summer, when the weather conditions facilitating outdoor activities.

Most of the deceased bats were male adult (n = 29/71, 40.8%) or subadult (n = 28/71, 39.4%). Juveniles and subadults have been reported to be more exposed to predation as they are smaller and easier to subdue and handle. Moreover, they can accidentally fall from the roost and get traumatized [30]. These results differed from those recorded by Mühldorfer et al. [20] and Ancillotto et al. [30], who reported that adult females were more often prayed or affected by disease, probably correlating with the maternal season.

Four *Hypsugo savii* bats also showed free nematodes in thoracic/abdominal cavities morphologically classified as *Litomosoides* spp. Filarial nematodes (*Onchocercidae*) of the genus *Litomosoides* spp. are hemoparasites and they have already been reported in the thoracic and/or abdominal cavities of marsupials, rodents, and bats of the Nearctic and Neotropical regions [31]. To the author’s knowledge, *Litosomoides* spp. has been recorded in the Italian bats since the middle of the last century even though limited data are available on the different *Litomosoides* species isolated from these bats [32]. Histologically, two of the four bats presented moderate and diffuse white pulp depletion and one of them also showed multifocal membranous glomerulopathy. No previous studies are available investigating the incidence of *Litosomoides* spp. infestation and the histopathological correlated lesions in *Hypsugo savii* bats. However, these lesions could be attributed to a potential immunopathogenic glomerular damage due to an antigenic effect of microfilariae similar to what has been already reported in dogs [33]. Further studies are needed to clarify the clinical significance of this parasite and its potential correlations with the observed histopathological lesions.

As far as histopathological findings are concerned, it is important to state that most of the samples were obtained from frozen animals. It is well known that freezing and dethawing can provoke different type of histological artifacts, mainly represented by loss of staining, extracellular fluid accumulation, cell shrinkage, fractures, haemolysis, and hematin formation. However, the freezing process still allow an adequate histological evaluation of the samples [34]. In fact, pneumonia of varying degrees was the most common lesion observed in all the species (n = 24/71, 33.8%). Particularly, interstitial pneumonia was characterized by lymphoplasmacytic infiltrates with bronchus-associated lymphoid tissue (BALT) activation compatible with an underlying infectious aetiology. However, in most of the affected lungs, neither infectious agents nor viral inclusion bodies were detected during microscopic examination. These results are in accordance with Mühldorfer et al. [19], which is the only study available evaluating histopathological findings in European bats. However, also Hajkova et al. [21] reported frequent macroscopical pulmonary lesions in bats in Czech Republic. Bats are excellent flyers in terms of speed, distance and maneuverability but flight is a very energetically demanding form of locomotion [35]. Thus, bat lungs have developed unique morphological, physiological and biochemical properties to enhance the uptake, transfer and utilization of oxygen for high aerobic capacities [35]. In fact, bats have remarkably big lungs that occupy a large proportion of the coelomic cavity and a relatively larger respiratory surface area compared to non-flying mammals [35]. Similar to birds, their large respiratory surface, their thin blood- gas barrier and their greater pulmonary capillary blood volume highly increase the respiratory efficiency, but it might also predispose bat lungs to injury from environmental toxicants and pathogens [35]. It has also been reported that the lack of exercise in captive or hospitalized bats might contribute to pulmonary disease, even though the underlying mechanisms are still unknown [29]. Two bats with lymphoplasmacytic bronchopneumonia were also positive for *poxvirus* isolation. No previous studies are available investigating histological lesions in lungs after *poxvirus* infection in bats. To the author’s knowledge, only Baker et al. [36] reported tenosynovitis, osteoarthritis or nodular skin lesions in bats with poxvirus infection. Thus, further studies are needed to clarify if there is causality between the *poxvirus* infection and the pneumonia recorded in the animals of the present study.

Patagium/skin dermatitis was the second most frequent histopathological finding. As already mentioned above, patagium lesions are likely to occur in bats and they can easily get infected by mixed bacterial populations (e.g., *Streptococcus* spp., *Staphylococcus* spp. etc.) [19]. Moreover, bat mites (e.g., *Notoedres muris, Demodex spp.*) can also cause severe dermatitis in bats [37] However, in the present study no mites were found in the skin/patagium samples and the lack of eosinophils permit to rule out parasitic dermatitis. Similarly, dermatitis can also be caused by fungi, especially *Geomyces destructans*, which cause the white nose syndrome in bats [38]. In fact, *G. destructans* initially grows on the skin’s surface of the wing and tail membranes and then progresses by invading the underlying tissues, where hyphae can form cup-like epidermal erosions and invade hair follicles, sebaceous glands, and the nearby connective tissue [38] To date, no fungal hyphae were observed at the histological examination of skin/patagium samples as confirmed by the absence of PAS and Gomori’s – Grocott staining positivity, excluding fungal dermatitis.

Moreover, liver steatosis was observed (n = 6/71, 8.4%). This lesion can be attributed to an unbalanced diet and overfeeding which is likely to occur in captivity [29]. In fact, in captivity bats frequently received diets richer in fatty acids compared to their natural feed and that could lead to liver steatosis [28]. To date, liver steatosis is likely to occur in winter when bats are rescued during hibernation. This is due to the fact that bat’s metabolism changed to utilize fat storages during hibernation and when enter a rescue centre it is forced to rapidly adapt to regularly feeding, causing steatosis. However, in this study steatosis was not recorded in the bats rescued in winter, being present only in summer, spring and autumn.

Also, hepatitis (n = 8/71, 11.3%) and white pulp depletion (n = 7/71, 9.8%) were observed but the aetiology behind these pathological processes is still unknown.

Rhabdoviruses, including Lyssaviruses and *Vaprio ledantevirus*, were not detected in the examined bats. As the sample included a single *Eptesicus serotinus*, considered as the reservoir host for *Lyssavirus hamburg*, and we did not analyse individuals of other bat species known to maintain other lyssaviruses described in European bats (i.e. *Myotis daubentonii, M. natterei* or *Miniopterus schreibersii*), the lack of positive findings for lyssaviruses was expected and is consistent with similar studies performed in the country [39]. On the other hand, *Vaprio ledantevirus* has been found in bats, including *P. kuhlii* and *H. savii* that have been largely screened in our investigation. However, the low prevalence reported for this virus in the literature and the scarce knowledge on its ecology likely prevented its identification in our samples [11].

We also failed to detect alpha and betacoronaviruses that have been frequently reported in the considered bat species, including viruses with zoonotic potential, such as MERS-like coronaviruses described in both pipistrelle bats and *Eptesicus serotinus*, also in Italy [13, 14]. This discrepancy can be related to the small sample size or to the different geographical areas considered [14]. Also, it can be attributed to a different sampling season compared to previous surveys, as several bat viruses, including coronaviruses, tend to follow seasonal dynamics with peaks of infection after the weaning of pups [40].

Finally, *Kobuvirus* was not detected in any of the analysed samples. *Kobuvirus* genus included *Aichivirus*, which are well-known enteric pathogens in humans and they have also been identified in a wide variety of vertebrate hosts, including both domestic and wild animals [41]. Interestingly, *Kobuvirus* was firstly isolated from bat faeces in Vietnam in 2018 and then a kobu-like virus was also observed in bat guano in Texas [42, 43]. Recently, a bat *Aichivirus* was also sequenced in a *Pipistrellus pipistrellus* in Italy, probably suggesting the presence of this virus also in European bats [9] However, the role of bats in A*ichivirus* transmission is still unclear and further studies are needed to better clarify their origin and evolutionary patterns [9].

On the contrary, 12 bats out 71 showed PCR positivity for *orthoreovirus* (16.4%) in the intestine and/or lungs. This data is consistent with their high frequency reported in the bats from the Old World including several species in Europe and Italy [10, 16, 17]. Among these, MRV was frequently isolated from *Pipistrellus khulii* in Italy, showing phylogenetic relationship with strains causing severe diseases in humans [10, 44] Even if the pathogenicity of MRV is low, with clinical disease occurring in neonates and immunocompromised people, the fact that *Pipistrellus khulii* is common in urban areas and known for its close contact with humans poses a potential risk for zoonotic transmission [10]. Moreover, the majority of the MRV positivity was recorded in summer, being in accordance with the study previously conducted in Italy by Lelli et al. [10]. Considering microscopical lesions, in the present study the bats infected with MRV showed heterogenous microscopical lesions: hepatic steatosis and hepatitis (n = 2/12, 33.3%), non-suppurative interstitial pneumonia (n = 2/12, 16.7%), white pulp depletion in the spleen (n = 1/12; 8.3%), and non-suppurative interstitial nephritis (n = 1/12, 8.3%). These findings are partially in accordance with Kohl et al. [16]. However, in contrast with Kohl et al. [16] no signs of enteritis and white pulp hyperplasia were recorded in the present study. Also, Lelli et al. [10] did not record any associated pathological lesions indicative of infectious diseases. The heterogenicity of these results confirmed that further studies are needed to clarify the impact of MRV infection on bats.

Finally, poxviruses were isolated in 2 bats of less than one year of age (n = 2/71, 2.8%). *Poxviridae* are a large, diverse family of DNA viruses that can infect a wide range of vertebrates and invertebrates. To date, only a few documented detections of poxviruses have been described in bat populations in America, Africa, and Australia [45–48]. The partial sequencing classified our virus as *Hypsugopoxvirus*, firstly described in Italy in 2019 in an *Hypsugo savi* bat. In our study, we confirmed the infection in the *H. savii* and further describe *Hypsugopoxvirus* in *P. kuhlii*, broadening its host range. As nothing is known on its ecology it is not possible to determine whether this finding is associated to low species-specificity of the virus or to cross-species transmission between the two bat species, an event already recorded for poxviruses in other species, such as between the grey squirrel (*Sciurus carolinensis*) and the red squirrel (*Sciurus vulgaris*) in Great Britain [49]. Similarly, only few information are available on the pathology of poxviruses in bats, generically associated with tenosynovitis, osteoarthritis and nodular skin lesions containing intracytoplasmic inclusion bodies [36]. Similarly, in Italy Lelli at al. [15] recorded a pathological healing of a humerus fracture associated with osteomalacia and calcium deficiency in a bat infected with *Hypsugopoxvirus*, potentially responsible for the bone degeneration.

In this context, our finding of mild and multifocal lymphoplasmacytic pneumonia represent an unusual feature possibly associated with poxvirus infection. To date, no data are available about lung infections in bats caused by Poxviruses. In fact, Poxviruses have been generally associated to skin disease in several species. However, it has also been reported that humans and macaques infected by variola virus, monkeypox, cowpox, and vaccinia virus generally show flulike symptoms, being bronchopneumonia the most frequent and serious complication of the disease [50]. Also, a cowpox virus infection has been reported to cause necrotizing facial dermatitis and a systemic syndrome involving lungs in a cat [51]. Due to the peculiar potential association among poxviral infection and lung inflammation in the bats of the present study, further investigations are necessary to better clarify the role of these viruses in the pathogenesis of the observed bronchopneumonia.

In conclusion, the present study highlighted that trauma could be the main cause of death in the bats of Turin province (North-western Italy) associated with forelimb fractures. Among viral investigations only Orthoreoviruses and Poxviruses were detected with moderate to low positivity rate (16.4 and 2.8%). Further studies are still needed to clarify the role of viral pathogens in the determination of bat’s histological lesions.

## Materials and methods

### Animals

All bat species in Europe are strictly protected under the Council Directive 92/43/EEC of 21 May 1992 on the conservation of natural habitats and of wild fauna and by the Agreement on the Conservation of Populations of European Bats (www.eurobats.org). For this study, carcasses of deceased bats found in Turin province (Italy) between October 2017 and October 2019 were kindly provided by the Non-Conventional Animal Centre (C.A.N.C) of the Department of Veterinary Sciences, University of Torino, which is a reference centre for the rescue and care of sick wild animals in the North of Italy. All the hospitalized subadults and adult bats were fed with *Tenebrio molitor* larvae supplemented with calcium carbonate and vitamin D. Neonates and juveniles were fed with artificial milk (Royal Canin, Milan, Italy). On arrival, after the assessment of their nutrition status, the daily dose of larvae and milk was established. Bats died in care or euthanized for animal welfare reasons were immediately necropsied or stored at -80 °C until necropsy. Bats were euthanized by an overdose of inhalation agents (5% isofluorane) in an induction chamber [52]. All the bats were sexed based on the reproductive organs and age was determined on the basis of the body mass, presence of teeth, fur and the epiphyseal-diaphyseal fusion at the metacarpal-phalangeal joints using transillumination previously reported by Brunet-Rossinni [53]. Briefly, neonates showed sparse pelage and they are toothless. Subadults still present milk teeth and there is still cartilage in the epiphyseal regions of the metacarpal-phalangeal joints. On the contrary, adults have no milk teeth left and epiphyseal bones are fused with the diaphysis.

### Necropsy and histological analysis

All the bats were submitted to a full necropsy at the Department of Veterinary Science, University of Torino (Italy). Animals underwent a thorough external examination for recording any fracture/dislocation, skin or patagium lesions, and for examining all the external orifices (ears, eyes, nose, anus, and oral cavity). Briefly, the animal was put in dorsal recumbency on a clean surface, pinning the patagium and the feet to stabilize the body (Fig. 1a). After partial skinning and opening of the thorax and abdomen, organs were examined in situ in order to detect any changes in terms of position, size, or colour and register the presence of any abnormal masses (Fig. 1b and c). Samples of lung, heart, liver, spleen, small intestine, kidney, and brain were collected for histopathological examination (Fig. 1d). Tissues were fixed in 10% buffered formalin (Kaltek S.r.l., Italy) and after 7 days of fixation, they were routinely embedded in paraffin (Leica, Germany) wax blocks, sectioned at 5 μm thickness, mounted on glass slides, and stained with Haematoxylin & Eosin (HE)(MilliporeSigma, Merck, USA). In addition, special histological staining methods were used depending on microscopic findings to exclude the presence of *Geomyces destructans* (periodic acid Schiff – Kaltek, Italy - or Grocott’s - Gomori methenamine silver nitrate –MilliporeSigma, Merck, USA - stainings). The most probable cause of death was recorded based on the most serious injury, disease, or event that could have been fatal to the animal.

### Biomolecular and virological investigations

For viral isolation, all the samples of lung, small intestine and pool of spleen and liver were homogenized in Minimal essential medium (Mem) (1 g/10 ml) containing penicillin and streptomycin (dilution 1:100 in MEM) and clarified by centrifugation at 3750 rpm for 30 min. Samples were inoculated in confluent monolayers of VERO (African green monkey kidney cells) and MARC-145 (Foetal monkey kidney cells) using 24 wells plates, incubated at 37 °C with 5% CO2, and observed daily for 7 days to highlight the development of the cytopathic effect (CPE). In the absence of CPE, the cryolysate was sub-cultured twice into fresh monolayers.

Also, samples of small intestine, liver, spleen, lung, and brain were collected and stored at -20 °C for virus detection using molecular methods and to confirm the species through the sequencing of the Cytochrome Oxidase I (COI) gene, as previously described by De Benedictis et al. [54].

No isolation was attempted from brain samples to preserve tissue available to rule out *Lyssavirus* infection using molecular biology. This evaluation was performed in all deceased bats by the National Reference Centre for Rabies at the Istituto Zooprofilattico Sperimentale Delle Venezie (Legnaro, Italy), independently from the species and the presence of neurological clinical signs, using a pan-lyssavirus RT-PCR from brain samples, performed according to the literature as detailed in the Additional file 4 [55]. Viral RNA was extracted from clinical samples by using the NucleoSpin RNA, Mini kit for RNA purification (Macherey-Nagel, Duren, Germany) for the analyses on lyssaviruses and COI following the manufacturer’s instructions [55].

Secondly, RT-PCRs were performed on all the samples targeting viruses belonging to the viral families *Coronaviridae, Poxviridae, Reoviridae* (*Mammalian orthoreovirus* species) and *Rhabdoviridae* (*Vaprio ledantevirus species*). Qiasymphony® DSP virus/pathogen (Qiagen, Germany) was used to extract the genetic material following the manufacturer’s instructions.

For the detection of coronaviruses, a pan-coronavirus nested RT-PCR was employed, targeting the RNA-dependent RNA polymerase (RdRp) sequence and obtaining final PCR products of 440 bp [56].

For the identification of reoviruses, a nested RT-PCR specific for *Mammalian orthoreovirus* (MRV) (*Orthoreovirus* genus) was used as screening test, targeting L1, a highly conserved gene that codes for λ3-RNA-dependent RNA polymerase (RdRp) [57]. The samples tested positive were then subjected to a multiplex RT-PCR (S1 RT-PCR), which amplifies a specific region of the S1 gene able to discriminate among the three serotypes of MRV [58].

For the detection of poxviruses, two pan-chordopoxvirus traditional PCR assays were used to detect poxviruses with low GC content and poxviruses with high GC content. For the low GC PCR the primers used were LGC F targeting insulin metalloproteinase-like protein gene and LGC R targeting intracellular mature virion membrane protein gene (IMV) with an amplicon size of 220 bp. For the high GC PCR the primers used are HGC F and HGC R, both targeting the RNA polymerase subunit gene with an amplicon size of 630 bp [59].

The presence of *Vaprio ledanteviru*s (genus: *Ledantevirus*, family: *Rhabdoviridae*), an emerging rhabdovirus recently detected from insectivorous bats in Northern Italy, was also investigated. For this purpose, a one-step end-point RT-PCR which amplify a highly conserved region of 350 bp of the ORF-6 gene, encoding the RNA-dependent RNA-polymerase (L protein), was performed [11].

Small intestinal and faecal samples were also submitted to Kobuviruses screening by SYBR Green one step Real time RT-PCR at National Reference Centre for Wild Animal Diseases (CeRMAS, Italy). Universal primers designed to amplify a 217-bp region of the 3D gene of all members of the genus *Kobuvirus* were used [60].

In every PCR run, negative and positive controls were included in addition to the samples. Specifically, DNA/RNA free water was used as negative control, while specific inactivated viral antigens were used as positive controls. In particular, for Panlyssavirus a laboratory strain of Rabies virus (CVS) was used as positive control. PCR protocols used in the study are reported in Table 2 and in Additional file 4.

The PCR products were then sequenced via the Sanger method and assembled in contigs using the Lasergene sequencing analysis software package (DNASTAR, Madison, WI, USA). Afterwards, the resulting sequences were compared to the GenBank database with BLAST using MEGA XI software (https://www.megasoftware.net/ accessed on 8 December 2022).

**Table 2:** PCR methods used for Coronavirus, Reovirus, Poxvirus, Rhabdovirus and Kobuvirus detection

Virus	PCR format	Primer	Sequence (5′→3′)	MRV specificity	References
*Lyssavirus*	RT-PCR	RabForPyro	AACACYYCTACAATGGA	n/a	[55]
		RabRevPyro 1	TCCAATTNGCACACATTTTGTG	n/a	
		RabRevPyro 2	TCCARTTAGCGCACATYTTATG	n/a	
		RabRevPyro 3	TCCAGTTGGCRCACATCTTRTG	n/a	
*Coronavirus*	Two-step RT PCR	Chu11-F1	GGKTGGGAYTAYCCKAARTG	n/a	[56]
		Chu11-R1	TGYTGTSWRCARAAYTCRTG	n/a	
	Nested PCR	Chu11-F2	GGTTGGGACTATCCTAAGTGTGA	n/a	
		Chu11-R2	CCATCATCAGATAGAATCATCAT	n/a	
*Mammalian orthoreovirus*	RT-PCR	L1-rv5F	GCATCCATTGTAAATGACGAGTCTG	All types	[57, 58]
		L1-rv6R	CTTGAGATTAGCTCTAGCATCTTCTG		
		L1-rv7F	GCTAGGCCGATATCGGGAATGCAG	All types	
		L1-rv8R	GTCTCACTATTCACCTTACCAGCAG		
		S1-R1F	GGAGCTCGACACAGCAAATA	Type 1	
		S1-R1R	GATGATTGACCCCTTGTGC		
		S1-R2F	CTCCCGTCACGGTTAATTTG	Type 2	
		S1-R2R	GATGAGTCGCCACTGTGC		
		S1-R3F	TGGGACAACTTGAGACAGGA	Type 3	
		S1-R3R	CTGAAGTCCACCRTTTTGWA		
*Poxvirus*	PCR	LGC F	ACACCAAAAACTCATATAACTTCT	n/a	[59]
		LGC R	CCTATTTTACTCCTTAGTAAATGAT	n/a	
		HGC F	CATCCCCAAGGAGACCAACGAG	n/a	
		HGC R	TCCTCGTCGCCGTCGAAGTC	n/a	
*Rhabdovirus* (*Vaprio ledantevirus*)	RT-PCR	IZSLER-VAPV F	TTGTTCCTCTGTTCAGCGGTC	n/a	[11]
		IZSLER-VV R	TCCGCCTAATTGTCCATTCC	n/a	
*Kobuvirus*	Real Time RT-PCR	UNIV-Kobu F	TGGAYTACAAG(/R)TGTTTTGATGC	n/a	[60]
		UNIV-Kobu R	ATGTTGTTRATGATGGTGTTGA	n/a	

Abbreviations: n/a not applicable; RT-PCR: reverse transcription polymerase chain reaction. Primers have been named according to original publications

### Statistical analysis

Statistical analysis was performed using R software version 4.0.4 (R Foundation for Statistical Computing, Vienna, Austria; http://www.r-project.org.). Data were described by bat species (*H.savii; P. khulii, P. nathusii, M. bechsteinii, M. mystacinus, P. macrobullaris, V. murinus, E.serotinus, T. tadarida*), sex (male and female), age (1: neonates, 2: juveniles, 3: subadults, and 4: adults) and season (winter, spring, summer and autumn) using the number of animals (n) and its percentage (%). For reporting the gross, histopathological, and virological findings the variable “age” was recategorized into two categories: <1 year (neonates, juveniles, and subadults) and ≥ 1 year (adults). Also, the variable “bat species” was recategorized and all the species represented by only 1 animal were grouped in a new variable called “Other species”. The statistical analysis code using for the present study has been uploaded in the GitHub repository (bit.ly/40JiAyF).

### Electronic supplementary material

Below is the link to the electronic supplementary material.

Supplementary Material 1

Supplementary Material 2

Supplementary Material 3

Supplementary Material 4

## Data Availability

The datasets generated and/or analysed during the current study are available in the BanKit GeneBank repository, under these GenBank accession numbers OR339562, OR339563, OR339564, OR339565, OR339566, OR339567, OR339568, OR339569, OR339570, OR339571, OR339572, OR339573, OR339574.

## Declarations

### ARRIVE guidelines

The study is reported in accordance with ARRIVE guidelines.
