# Supplementary material for: Main causes of death of free-ranging bats in Turin province (North-Western Italy): gross and histological findings and emergent virus surveillance
Source: BMC Vet Res. 2023 Oct 11;19:200. doi: 10.1186/s12917-023-03776-0 (PMC10566203; doi:10.1186/s12917-023-03776-0)
Supplement: Supplementary file 1 — Supplementary Material 1 [file 12917_2023_3776_MOESM1_ESM.docx]

**Additional file 1. Causes of mortality and macroscopical findings detected in the bats of the present study (n=71).**

|  |  | Species | | | | Age | | Sex | |
| --- | --- | --- | --- | --- | --- | --- | --- | --- | --- |
|  | Tot (%) | *H. savii* | *P. khulii* | *P. nathusii* | Other | <1 year | Adult | Male | Female |
| Causes of deaths |  |  |  |  |  |  |  |  |  |
| Trauma | 35  (49.3%) | 16  (45.7%) | 16  (45.7%) | 2  (5.7%) | 1  (2.9%) | 16 (45.7%) | 19  (54.3%) | 27  (77.1%) | 8  (22.9%) |
| Predation | 5  (7.0%) | 1  (20.0%) | 4  (80.0%) | 0  (0.0%) | 0  (0.0%) | 1  (20.0%) | 4  (80.0%) | 2  (40.0%) | 3  (60.0%) |
| Emaciation | 13  (18.3%) | 4  (30.8%) | 7  (53.8%) | 0  (0.0%) | 2  (15.4%) | 13  (100.0%) | 0  (0.0%) | 8  (61.5%) | 5  (38.5%) |
| Unknown | 18  (25.4%) | 7  (38.9%) | 7  (38.9%) | 1  (5.5%) | 3  (16.7%) | 12  (66.7%) | 6  (33.3%) | 8  (44.4%) | 10  (55.6%) |
| Macroscopical lesions |  |  |  |  |  |  |  |  |  |
| Fractures | 15  (21.1%) | 6  (40.0%) | 8  (53.3%) | 1  (6.7%) | 0  (0.0%) | 8  (53.3%) | 7  (46.7%) | 11  (73.3%) | 4  (26.7%) |
| Patagium/skin lesions^1^ | 23  (32.4%) | 12  (52.2%) | 8  (34.8%) | 2  (8.7%) | 1  (4.3%) | 11  (47.8%) | 12  (52.2%) | 15  (65.2%) | 8  (34.8%) |
| Nematods in thorax/abdomen | 4  (5.6%) | 4  (100.0%) | 0  (0.0%) | 0  (0.0%) | 0  (0.0%) | 2  (50.0%) | 2  (50.0%) | 4  (100.0%) | 0  (0.0%) |
| Liver petecchiae and abdominal haemorrhages | 1  (1.4%) | 1  (100.0%) | 0  (0.0%) | 0  (0.0%) | 0  (0.0%) | 1  (100.0%) | 0  (0.0%) | 1  (100.0%) | 0  (0.0%) |
| Spleen decolouration | 1  (1.4%) | 0  (0.0%) | 1  (100.0%) | 0  (0.0%) | 0  (0.0%) | 1  (100.0%) | 0  (0.0%) | 0  (0.0%) | 1  (100.0%) |
| Gastric distention | 10 (14.1%) | 5  (50.0%) | 3  (30.0%) | 1  (10.0%) | 1  (10.0%) | 5  (50.0%) | 5  (50.0%) | 7  (70.0%) | 3  (30.0%) |
| Pneumonia | 3  (4.2%) | 1  (33.3%) | 2  (66.7%) | 0  (0.0%) | 0  (0.0%) | 2  (66.7%) | 1  (33.3%) | 1  (33.3%) | 2  (66.7%) |
| Diaphrammatic hernia | 1  (1.4%) | 0  (0.0%) | 1  (100.0%) | 0  (0.0%) | 0  (0.0%) | 1  (100.0%) | 0  (0.0%) | 1  (100.0%) | 0  (0.0%) |
| Non-significant findings | 27 (38.0%) | 7  (25.9%) | 15  (55.6%) | 1  (3.7%) | 4  (14.8%) | 18  (66.7%) | 9  (33.3%) | 10  (37.0%) | 17  (63.0%) |

^1^ patagium lacerations, necrotizing dermatitis, alopecia, cutaneous/subcutaneous abscesses
