# Supplementary material for: Main causes of death of free-ranging bats in Turin province (North-Western Italy): gross and histological findings and emergent virus surveillance
Source: BMC Vet Res. 2023 Oct 11;19:200. doi: 10.1186/s12917-023-03776-0 (PMC10566203; doi:10.1186/s12917-023-03776-0)
Supplement: Supplementary file 2 — Supplementary Material 2 [file 12917_2023_3776_MOESM2_ESM.docx]

**Additional file 2. Main histopathological lesions detected in the bats of the present study (n=71)**

|  |  | Species | | | | Age | | Sex | |
| --- | --- | --- | --- | --- | --- | --- | --- | --- | --- |
|  | N (%) | *H. savii* | *P. khulii* | *P. nathusi* | Other | <1 year | Adult | Male | Female |
| Microscopical lesions |  |  |  |  |  |  |  |  |  |
| Lung | 24 (33.8%) | 11  (45.8%) | 10  (41.7%) | 1  (4.2%) | 2  (8.3%) | 19  (79.2%) | 5  (20.8%) | 16  (66.7%) | 8  (33.3%) |
| Liver | 12 (16.9%) | 4  (33.4%) | 6  (50.0%) | 1  (8.3%) | 1  (8.3%) | 7  (58.3%) | 5  (41.7%) | 7  (58.3%) | 5  (41.7%) |
| Spleen | 10 (14.1%) | 8  (80.0%) | 2  (20.0%) | 0  (0.0%) | 0  (0.0%) | 7  (70.0%) | 3  (30.0%) | 8  (80.0%) | 2  (20.0%) |
| Kidney | 3  (4.2%) | 2  (66.7%) | 1  (33.3%) | 0  (0.0%) | 0  (0.0%) | 1  (33.3%) | 2  (66.7%) | 3  (100.0%) | 0  (0.0%) |
| Patagium/skin | 23  (32.4%) | 12  (52.2%) | 8  (34.8%) | 2  (8.7%) | 1  (4.3%) | 11  (47.8%) | 12  (52.2%) | 15  (65.2%) | 8  (34.8%) |
| Intestine | 3  (4.2%) | 0  (0.0%) | 2  (66.7%) | 1  (33.3%) | 0  (0.0%) | 1  (33.3%) | 2  (66.7%) | 1  (33.3%) | 2  (66.7%) |
| Brain | 0  (0.0%) | 0  (0.0%) | 0  (0.0%) | 0  (0.0%) | 0  (0.0%) | 0  (0.0%) | 0  (0.0%) | 0  (0.0%) | 0  (0.0%) |
| Heart | 0  (0.0%) | 0  (0.0%) | 0  (0.0%) | 0  (0.0%) | 0  (0.0%) | 0  (0.0%) | 0  (0.0%) | 0  (0.0%) | 0  (0.0%) |
| Non- significant lesions | 31 (43.7%) | 9  (29.0%) | 17  (54.8%) | 1  (3.3%) | 4  (12.9%) | 14  (45.2%) | 17  (54.8%) | 17  (54.8%) | 14  (45.2%) |
