# Supplementary material for: Main causes of death of free-ranging bats in Turin province (North-Western Italy): gross and histological findings and emergent virus surveillance
Source: BMC Vet Res. 2023 Oct 11;19:200. doi: 10.1186/s12917-023-03776-0 (PMC10566203; doi:10.1186/s12917-023-03776-0)
Supplement: Supplementary file 3 — Supplementary Material 3 [file 12917_2023_3776_MOESM3_ESM.docx]

|  |  | Species | | | | Age | | Sex | | Season | | | |  |
| --- | --- | --- | --- | --- | --- | --- | --- | --- | --- | --- | --- | --- | --- | --- |
|  | N (%) | *H. savii* | *P. khulii* | *P. nathusi* | Other | <1 year | Adult | Male | Female | Spring | Summer | Autumn | Winter |  |
| Microscopical lesions |  |  |  |  |  |  |  |  |  |  |  |  |  |  |
| Lung | 24 (33.8%) | 11  (45.8%) | 10  (41.7%) | 1  (4.2%) | 2  (8.3%) | 19  (79.2%) | 5  (20.8%) | 16  (66.7%) | 8  (33.3%) | 2  (8.3%) | 18  (75.0%) | 4  (16.7%) | 0  (0.0%) |  |
| Liver | 12 (16.9%) | 4  (33.4%) | 6  (50.0%) | 1  (8.3%) | 1  (8.3%) | 7  (58.3%) | 5  (41.7%) | 7  (58.3%) | 5  (41.7%) | 1  (8.3%) | 10  (83.4%) | 1  (8.3%) | 0  (0.0%) |  |
| Spleen | 10 (14.1%) | 8  (80.0%) | 2  (20.0%) | 0  (0.0%) | 0  (0.0%) | 7  (70.0%) | 3  (30.0%) | 8  (80.0%) | 2  (20.0%) | 0  (0.0%) | 7  (70.0%) | 3  (30.0%) | 0  (0.0%) |  |
| Kidney | 3  (4.2%) | 2  (66.7%) | 1  (33.3%) | 0  (0.0%) | 0  (0.0%) | 1  (33.3%) | 2  (66.7%) | 3  (100.0%) | 0  (0.0%) | 0  (0.0%) | 1  (33.3%) | 1  (33.3%) | 1  (33.3%) |  |
| Patagium/skin | 23  (32.4%) | 12  (52.2%) | 8  (34.8%) | 2  (8.7%) | 1  (4.3%) | 11  (47.8%) | 12  (52.2%) | 15  (65.2%) | 8  (34.8%) | 3  (13.0%) | 14  (60.8%) | 4  (17.4%) | 2  (8.7%) |  |
| Intestine | 3  (4.2%) | 0  (0.0%) | 2  (66.7%) | 1  (33.3%) | 0  (0.0%) | 1  (33.3%) | 2  (66.7%) | 1  (33.3%) | 2  (66.7%) | 0  (0.0%) | 2  (66.7%) | 1  (33.3%) | 0  (0.0%) |  |
| Non- significant lesions | 31 (43.7%) | 9  (29.0%) | 17  (54.8%) | 1  (3.3%) | 4  (12.9%) | 14  (45.2%) | 17  (54.8%) | 17  (54.8%) | 14  (45.2%) | 2  (6.4%) | 18  (58.1%) | 7  (22.6%) | 4  (12.9%) |  |

**Additional file 3.** Main histopathological lesions detected in the bats from Turin province in 2018-2021 (n=71).
