# Supplementary material for: Main causes of death of free-ranging bats in Turin province (North-Western Italy): gross and histological findings and emergent virus surveillance
Source: BMC Vet Res. 2023 Oct 11;19:200. doi: 10.1186/s12917-023-03776-0 (PMC10566203; doi:10.1186/s12917-023-03776-0)
Supplement: Supplementary file 4 — Supplementary Material 4 [file 12917_2023_3776_MOESM4_ESM.docx]

**PCR PROTOCOLS**

**Pan-lyssavirus RT-PCR**

Viral RNA was extracted from clinical samples a by using NucleoSpin RNA, Mini kit for RNA purification (Macherey-Nagel, Duren, Germany), according to manufacturer’s instructions. Briefly, 100 μl of sample suspension was used for the extraction, and RNA was eluted in a final volume of 60 μl and stored at −80°C.

Pan-lyssavirus RT-PCR was performed as previously described (56), with slight modifications. Briefly, the lyssavirus-specific primer set, designed on a relatively conserved region of the N gene, allowed for the amplification and further sequencing of a 603-bp expected product. None of the primers used was biotinylated as no further pyrosequencing technique was planned to be applied.

).

TABLE: Primer sequences targeting the lyssavirus nucleoprotein gene 3′ terminus

| **Primer** | **Sequence (5′–3′)** | **Position*^a^*** |
| --- | --- | --- |
| RabForPyro | AACACYYCTACAATGGA | 59–75 |
| RabRevPyro-biot 1 | TCCAATTNGCACACATTTTGTG | 662–641 |
| RabRevPyro-biot 2 | TCCARTTAGCGCACATYTTATG | 662–641 |
| RabRevPyro-biot 3 | TCCAGTTGGCRCACATCTTRTG | 662–641 |

The reverse primer mixture was composed by an equal concentration of all three primers, allowing for better control of the ratio of the different degenerated primers in the final mixture.

The final primer concentration applied to the PCR was 400 nM. 2.5 microliters of isolated RNA was added to 20 μl of master mix, with a final volume of 25 μl. The following protocol was used: 30 min at 50°C and 15 min at 95°C followed by 45 cycles at 94°C for 30 s, 52°C for 30 s, and 72°C for 40 s. Amplicons were detected by gel electrophoresis using 7% silver-stained polyacrylamide gel or using a QIAxcel Advanced DNA/RNA Analyzer (Qiagen, Hilden, Germany).

**Pan-coronavirus nested RT-PCR targeting RpRd gene**

The nucleic acid was extracted using the QIAsymphony DSP Virus\Pathogen mini kit (Qiagen, Germany) following the manufacturer’s instruction.

Assay was performed with the SuperScript III One-Step RT PCR System (Invitrogen). The primers used were Chu11-F1 (5’-GGKTGGGAYTAYCCKAARTG-3’) and Chu11-R1 (5’-TGYTGTSWRCARAAYTCRTG-3’) for the first PCR step. The second PCR step was performed with the HotStartaq MM (Qiagen, Hilden, Germany) and the primers used were Chu11-F2 (5’-GGTTGGGACTATCCTAAGTGTGA-3’) and Chu11-R2 (5’-CCATCATCAGATAGAATCATCAT-3’). The reaction mixture contained 0.2 μM of sense and antisense primer and 5 μl of RNA template in a final volume of 25 μl. The thermal cycling conditions for the first PCR step consisted of 30 min at 50° C for reverse transcription, 2 min at 94° C for the initial enzyme activation followed by 40 thermal cycles (94° C for 20 s, 48° C for 30 s, 72° C for 50 s), with a final elongation step of 68° C for 5 min. The second PCR step was performed with the same cycling condition. Amplicons were visualized on 2% agarose electrophoretic gels, which were stained with EuroSafe Fluorescent Nucleic Acid Stain (Euroclone, Milan, Italy) used at a 1× concentration. The final amplified product consit of 440 pb (57).

**Nested RT-PCR for Mammalian orthoreovirus (MRV) detection targeting L1 gene**

The nucleic acid was extracted using the QIAsymphony DSP Virus\Pathogen mini kit (Qiagen, Germany) following the manufacturer’s instruction.

Assay was performed with the QIAGEN® OneStep RTPCR master mix (Qiagen, Hilden, Germany).

The primers used were L1-rv5F (5’-GCATCCATTGTAAATGACGAGTCTG-3’) and

L1-rv6R (5’CTTGAGATTAGCTCTAGCATCTTCTG-3’) for the first PCR step. The second PCR step was performed with the HotStartaq MM (Qiagen, Hilden, Germany). The primers used were L1-rv7F (5’-GCTAGGCCGATATCGGGAATGCAG-3’) and L1-rv8R (5’-GTCTCACTATTCACCTTACCAGCAG-3’). The reaction mixture contained contained 0.6 μM (first step) and 0.2 μM (second step) of sense and antisense primer and 5 μl of denaturated RNA template (95° for 5’) in a final volume of 25 μl. The thermal cycling conditions for both PCR steps consisted of 30 min at 50° C for reverse transcription, 15 min at 95 °C for the initial enzyme activation followed by 40 thermal cycles (94° C for 30 s, 56° C for 30 s, 72° C for 1 min), with a final elongation step of 72° C for 10 min. Amplicons were visualized on 2% agarose electrophoretic gels, which were stained with EuroSafe Fluorescent Nucleic Acid Stain (Euroclone, Milan, Italy) used at a 1× concentration. The final amplified product consists of 344 pb (58).

**Multiplex RT-PCR for Mammalian orthoreovirus (MRV) typing targeting S1 gene**

The nucleic acid was extracted using the QIAsymphony DSP Virus\Pathogen mini kit (Qiagen, Germany) following the manufacturer’s instruction.

Assay was performed with the QIAGEN® OneStep RTPCR master mix (Qiagen, Hilden, Germany). The primers used were S1-R1F 5’-GGAGCTCGACACAGCAAATA-3’ and S1-R1R 5’-GATGATTGACCCCTTGTGC-3’ for MRV type 1, 5’-S1-R2F CTCCCGTCACGGTTAATTTG-3’ and S1-R2R 5’-GATGAGTCGCCACTGTGC-3’ for MRV type 2, S1-R3F 5’-TGGGACAACTTGAGACAGGA-3’ and S1-R3R 5’-CTGAAGTCCACCRTTTTGWA-3’ for MRV type 3.The reaction mixture contained 0.6 μM of sense and antisense primer and 5 μl of denaturated RNA template (95° for 5’) in a final volume of 25 μl. The thermal cycling conditions consisted of 30 min at 50 °C for reverse transcription, 15 min at 95 °C for the initial enzyme activation followed by 40 thermal cycles (94° C for 30 s, 54° C for 45 s, 72° C for 1 min), with a final elongation step of 72° C for 10 min. Amplicons were visualized on 2% agarose electrophoretic gels, which were stained with EuroSafe Fluorescent Nucleic Acid Stain (Euroclone, Milan, Italy) used at a 1× concentration. The final amplified product consists of 505 pb for MRV type1, 394 pb for MRV type2 and 326 pb for MRV type3 (59).

**Pan-chordopoxvirus PCR – Low GC targeting RNA polymerase subunit gene**

The nucleic acid was extracted using the QIAsymphony DSP Virus\Pathogen mini kit (Qiagen, Germany) following the manufacturer’s instruction.

Assay was performed with the HotStartaq MM (Qiagen, Hilden, Germany). The primers used were LGC F 5’-ACACCAAAAACTCATATAACTTCT-3’ and LGC R 5’CCTATTTTACTCCTTAGTAAATGATChu11-3’. The reaction mixture contained 0.2 μM of sense and antisense primer and 5 μl of RNA template in a final volume of 25 μl. The thermal cycling conditions consisted of 15 min at 94° C for the initial enzyme activation followed by 35 thermal cycles (94° C for 30 s, 53° C for 30 s, 72° C for 15 s), with a final elongation step of 72° C for 10 min. Amplicons were visualized on 2% agarose electrophoretic gels, which were stained with EuroSafe Fluorescent Nucleic Acid Stain (Euroclone, Milan, Italy) used at a 1× concentration. The final amplified product consists of 220 pb (60).

**Pan-chordopoxvirus PCR – High GC targeting IMV gene**

The nucleic acid was extracted using the QIAsymphony DSP Virus\Pathogen mini kit (Qiagen, Germany) following the manufacturer’s instruction.

Assay was performed with the HotStartaq MM (Qiagen, Hilden, Germany). The primers used were HGC F 5’-CATCCCCAAGGAGACCAACGAG-3’ and HGC R 5’TCCTCGTCGCCGTCGAAGTCLGC F-3’. The reaction mixture contained 0.2 μM of sense and antisense primer and 5 μl of RNA template in a final volume of 25 μl. The thermal cycling conditions consisted of 15 min at 94° C for the initial enzyme activation followed by 35 thermal cycles (94° C for 30 s, 60° C for 30 s, 72° C for 45 s), with a final elongation step of 72° C for 10 min. Amplicons were visualized on 2% agarose electrophoretic gels, which were stained with EuroSafe Fluorescent Nucleic Acid Stain (Euroclone, Milan, Italy) used at a 1× concentration. The final amplified product consists of 630 pb (60).

**One-step end-point RT-PCR for Vaprio ledantevirus targeting ORF6 gene**

The nucleic acid was extracted using the QIAsymphony DSP Virus\Pathogen mini kit (Qiagen, Germany) following the manufacturer’s instruction.

Assay was performed with the QIAGEN® OneStep RTPCR master mix (Qiagen, Hilden, Germany). The primers used were IZSLER-VAPV F (5′- TTG TTC CTC TGT TCA GCG GTC -3′) and IZSLER-VV R (5′- TCC GCC TAA TTG TCC ATT CC -3′). The reaction mixture contained 0.6 μM of sense and antisense primer and 5 μl of the RNA template in a final volume of 25 μl. The thermal cycling conditions consisted of 30 min at 50 °C for reverse transcription, 15 min at 95 °C for the initial enzyme activation and 7 touchdown thermal cycles (94 °C for 30 s; from 63 °C to 54 °C for 45 s; and 72 °C for 45 s). This was followed by 35 thermal cycles (94 °C for 30 s; 54 °C for 45 s; and 72 °C for 45 s), with a final elongation step of 72 °C for 5 min. Amplicons were visualized on 2% agarose electrophoretic gels, which were stained with EuroSafe Fluorescent Nucleic Acid Stain (Euroclone, Milan, Italy) used at a 1× concentration. The final amplified product consit of 350 pb (11).

**SYBR Green one step Real time RT-PCR for KOBUVIRUS**

The intestinal swabs were diluted in 1 ml of PBS and vigorously vortexed. Extraction was performed on 500 µl of sample in PBS, with QIAzol/chloroform method, according to QIAzol manufacturer’s instructions (Qiagen). The final RNA pellet was resuspended in 50 µl of RNase-free water.

The primers used were generic primers for Kobuvirus: UNIV-kobu-F (forward, 5′- TGGAYTACAAG(/R)TGTTTTGATGC, corresponding to nts 7491–7512 of U-1 virus) and UNIV-kobu-R (reverse, 5′- ATGTTGTTRATGATGGTGTTGA , corresponding to nts 7686–7707 of U-1 virus).

The reaction mixture contained 0.5 µl of SuperScript III Reverse ranscriptase/Platinum Taq DNA Polymerase Mix, 12.5 µl of 2X SYBR Green Reaction

Mix, 0.5 µl of ROX Reference Dye (SuperScript III Platinum SYBR Green qRT-PCR kit, Invitrogen), 5.5 µl of RNAse-free water, 0.5 µl of each 10 µM forward and reverse primer and 5 µl RNA.

The thermal cycling conditions consisted of 15 min at 50 °C for reverse transcription, followed by a step of 5 min at 95°C and 40 cycles consisting of 15 s at 94°C, 30 s at 58°C and 30 s at 72°C. At last, to increase test specificity, a melting stage was added, increasing the temperature from 60°C to 95°C at ramping increments of 0.3°C/s.

Fluorescence data were analysed for each sample by using the StepOne™ Software v2.3 (Thermofisher) and only samples whose fluorescence exceed the threshold with a C T ≤36 and simultaneously have a melting temperature between 86.3 and 87.6°C are considered positive (61).
